# Factors Affecting Patient Satisfaction in the Emergency Department: A Systematic Review

**DOI:** 10.7759/cureus.99509

**Published:** 2025-12-17

**Authors:** Mohammed Asiri, Ahmed S AlMohimeed, Moneef Almoneef

**Affiliations:** 1 Emergency Medicine, Prince Sultan Military Medical City, Riyadh, SAU; 2 Unaizah College of Medicine and Medical Sciences, Qassim University, Unaizah, SAU; 3 Medical Education, Prince Sultan Military Medical City, Riyadh, SAU

**Keywords:** emergency department, healthcare quality, patient experience, patient satisfaction, predictor of satisfaction

## Abstract

Patient satisfaction is widely recognized as a key indicator of healthcare quality and an essential measure of performance in emergency departments. As the emergency department represents the first point of contact for many patients, understanding the factors that influence satisfaction is crucial for improving care delivery. This systematic review aimed to assess patient satisfaction and identify the factors associated with satisfaction in the emergency department. A systematic search of PubMed and Google Scholar was conducted to identify relevant studies published between 2019 and 2022 using combinations of the keywords "Factors," "Affecting," "Patient," "Satisfaction," "Emergency," and "Department." Only original, full-text research articles addressing patient satisfaction in the emergency department were included. Of the 100 records initially identified, seven studies met the eligibility criteria and were included in the final analysis.

The included studies consisted of three cross-sectional studies, two retrospective studies, one case study, and one randomized controlled trial, conducted across Saudi Arabia, Portugal, Italy, Rotterdam, and Iran, with a total sample exceeding 3,491 emergency department patients. Overall patient satisfaction across the included studies ranged from relatively satisfied to completely satisfied. Key predictors of patient satisfaction included overall doctor satisfaction, perceived waiting time for triage, and meeting patient expectations. Additional influential factors included tangibles, assurance, reliability, responsiveness, and empathy. Shorter emergency department length of stay and the provision of general, medical, and practical information were associated with higher satisfaction, with oral communication and written leaflets being the preferred methods of information delivery.

Studies conducted in Saudi Arabia reported moderate levels of satisfaction with physician and nursing care, high preference for waiting time estimation, and relatively lower satisfaction with translation services, drug information, and pain management. These findings indicate that patient satisfaction in emergency departments is influenced by a combination of communication quality, service organization, and patient-provider interaction. Targeted interventions focusing on improving communication, managing waiting time expectations, and enhancing information delivery may contribute to improved patient satisfaction in emergency care settings.

## Introduction and background

Patient satisfaction is widely regarded as an important indicator of how well emergency departments deliver care and services [1]. It is defined as the patient’s opinion of the care received in comparison to what was expected [2]. In low- and middle-income countries, emergency care plays a crucial role in reducing preventable deaths and disability [3]. With increasing public awareness and knowledge of healthcare services, evaluating healthcare quality and improving patient satisfaction have become global health priorities for both providers and healthcare systems [4].

Patient satisfaction is a fundamental element of healthcare, particularly in emergency departments, which represent the patient’s primary point of entry into the healthcare system [5]. It is also considered one of the most important indicators of the effectiveness and quality of emergency care [6]. However, patient satisfaction is influenced by several factors, including patients’ health status, educational level, occupation, and socioeconomic background [7]. To address the conditions that lead to patient dissatisfaction, a range of interconnected factors contributing to patient satisfaction must be explored [8].

Furthermore, patient satisfaction has been shown to correlate strongly with continuity of care. Higher satisfaction levels influence patient adherence to treatment plans and increase the likelihood of returning to and receiving care from the same healthcare facility and professionals [9]. In addition to waiting time, which is considered a key determinant of satisfaction in emergency departments, clear explanations of laboratory results, medical conditions, and reasons for admission have been shown to significantly affect patient satisfaction [10]. Despite the importance of these factors, research assessing patient satisfaction with emergency care services in Saudi Arabia remains limited. Therefore, this systematic review was conducted to assess patient satisfaction and the factors related to patient satisfaction in the emergency department.

## Review

Method

Study Design

This systematic review was conducted in accordance with the PRISMA (Preferred Reporting Items for Systematic Reviews and Meta-Analyses) guidelines for systematic reviews and meta-analyses [11].

Search Strategy

Two electronic databases, PubMed and Google Scholar, were searched to identify studies relevant to the topic of factors affecting patient satisfaction in the emergency department. Only studies published between 2019 and 2022 were considered. The search process involved combinations of the following keywords: “Factors”, “Affecting”, “Patient”, “Satisfaction”, “Emergency”, and “Department.” The initial search resulted in the identification of potentially relevant articles, and all retrieved titles were subsequently screened.

Eligibility Criteria

**Figure 1:**
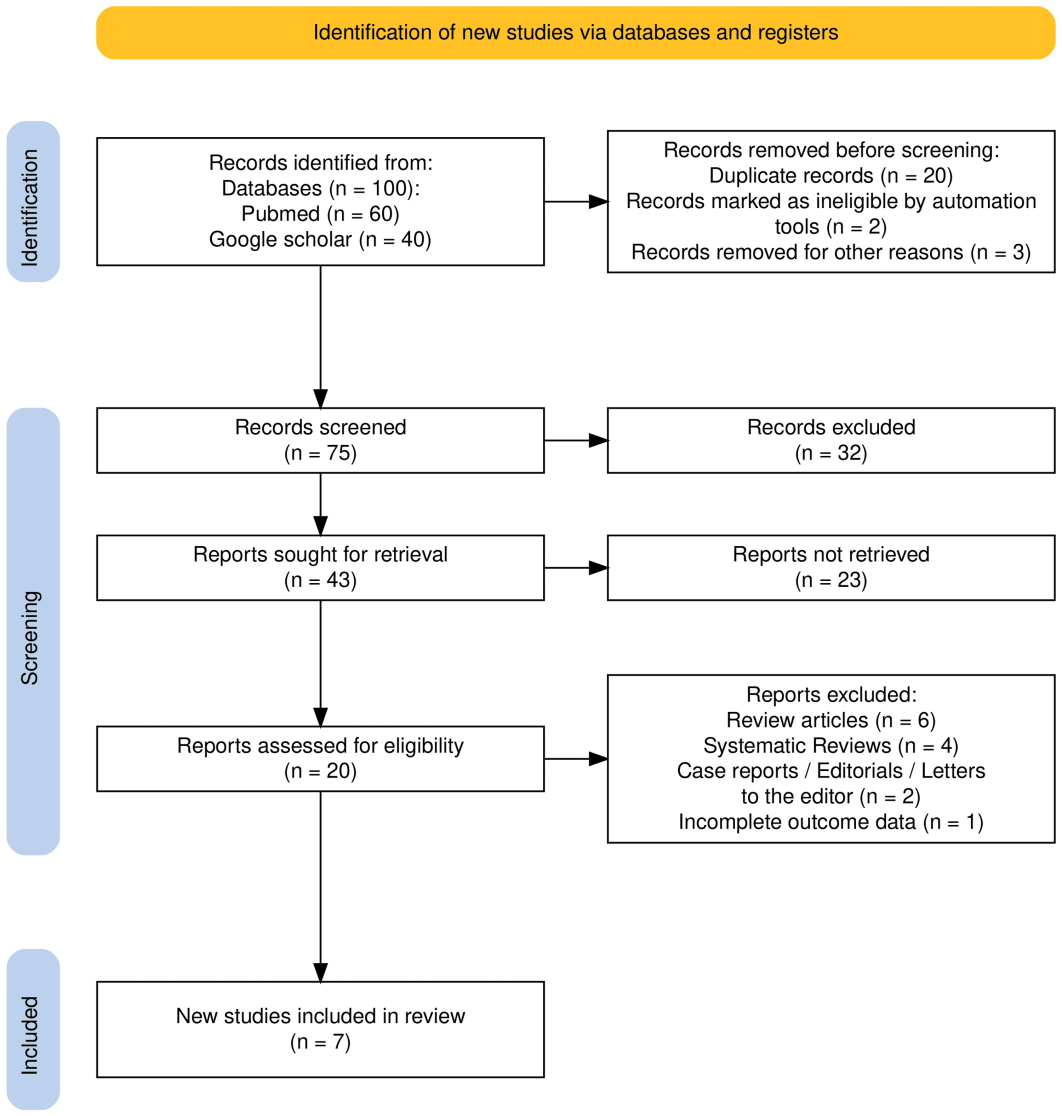
PRISMA flow diagram illustrating the study selection process for the systematic review. PRISMA: Preferred Reporting Items for Systematic Reviews and Meta-Analyses. Referenece: [12].

Studies were included if they were original research articles, were written in English, and addressed factors influencing patient satisfaction in the emergency department. During the initial screening phase, titles and abstracts were reviewed to assess relevance and publication period. Case reports, editorials, letters to the editor, and review articles were excluded. In the final stage, full-text articles were assessed to remove duplicate publications, non-full-text publications, and studies with incomplete or overlapping data. The detailed study selection process is summarized in the PRISMA flow diagram (Figure 1) [12].

Study Selection

Study selection was performed in multiple stages. After the initial title screening, abstracts were reviewed to determine eligibility based on the predefined criteria. Full-text articles were then assessed for final inclusion. Only studies that met all inclusion criteria were included in the final analysis.

Data Extraction

The full texts and abstracts of the selected studies were reviewed to extract relevant information. The extracted data were organized into a structured Excel spreadsheet (Microsoft Corporation, Redmond, Washington), where they were categorized and summarized to facilitate comparison across studies.

Data Analysis

The extracted data were analyzed descriptively. The final dataset was used to identify common themes and key factors influencing patient satisfaction in the emergency department.

Quality Assessment

A formal risk of bias or quality assessment tool was not applied, as the primary aim of this review was to provide a descriptive summary of the available literature on factors influencing patient satisfaction in the emergency department.

Results

Seven studies met the eligibility criteria for this systematic review [13-19] (Table 1). The included studies were published between 2019 and 2022, with two studies published in 2019 [15,19], two in 2020 [13,17], two in 2021 [14,18], and one in 2022 [16]. Regarding study design, three studies were cross-sectional [16,18,19], two were retrospective [13,15], one was a case study [14], and one was a randomized controlled trial [17].

**Table 1:** Overview of the included studies, including study design, setting, population, and main findings. ED: emergency department.

Author / Year	Country	Study Design	Sample Size	Objective	Main Findings
Abidova et al., 2020 [13].	Portugal	Cross-sectional	421	Identify predictors of patient satisfaction in ED	Communication clarity strongest predictor.
Stefanini et al., 2021 [14].	Italy	Observational	186	Examine complex interactions influencing satisfaction	Stress and perceived crowding influenced satisfaction.
Mandel et al., 2019 [15].	USA	Quasi-experimental	195	Evaluate music therapy in the ED	Music therapy improved satisfaction.
de Steenwinkel et al., 2022 [16].	Netherlands	Cross-sectional	200	Assess patient needs & information preferences	Tailored information increased satisfaction.
Alrajhi et al., 2020 [17].	Saudi Arabia	Cross-sectional	318	Assess the effect of waiting time estimates	Waiting time estimates improved satisfaction.
Abass et al., 2021 [18].	Saudi Arabia	Cross-sectional	512	Evaluate satisfaction in the teaching hospital ED	Moderate satisfaction; communication issues are notable.
Mohammadi-Sardo and Salehi, 2019 [19].	Iran	Cross-sectional	250	Assess ED satisfaction using SERVQUAL	Responsiveness and empathy gaps lowered satisfaction.

Two studies were conducted in Saudi Arabia [17,18], one in Portugal [13], one in Italy [14], and one in Rotterdam [16]. The total number of participants included across all studies exceeded 3,491 emergency department patients.

Two studies primarily assessed overall patient satisfaction in the emergency department [13,19], while two studies investigated predictors and variables associated with patient satisfaction [14,16]. Two additional studies examined patient satisfaction in relation to the impact of emergency department waiting time estimates [17,18]. One study evaluated the effect of music therapy services on patient satisfaction, stress, and pain among emergency department patients by comparing satisfaction scores between patients who received music therapy and those who did not [15].

Regarding overall patient satisfaction, two studies [13,16] reported that the mean satisfaction scores ranged from relatively satisfied to completely satisfied. In one study [16], most respondents expressed satisfaction with the medical and general information provided; however, satisfaction with useful information was lower. A comparative summary of findings across the included studies is presented in Table 2.

**Table 2:** Comparative summary of positive and negative factors influencing patient satisfaction across included study

Study	Positive Factors	Negative Factors	Effect on Satisfaction	Key Conclusion
Abidova et al., 2020 [15].	Clear communication, respectful staff	Long waiting time	Improved with strong communication	Communication strongest predictor
Stefanini et al., 2021 [16].	Reduced stress, supportive environment	Perceived crowding	Stress strongly impacted satisfaction	Psychological factors influential
Mandel et al., 2019 [17].	Music therapy reduced stress	Noise, discomfort	Improved with music therapy	Non-clinical interventions help
de Steenwinkel et al., 2022 [18].	Frequent updates, tailored information	Unclear explanations	Higher with tailored information	Information delivery crucial
Alrajhi et al., 2020 [19].	Waiting time estimates	Uncertainty, overcrowding	Higher when time was known	Expectation management matters
Abass et al., 2021 [20].	Courteous staff, clean environment	Weak communication	Moderate satisfaction	Communication gaps persistent
Mohammadi-Sardo and Salehi, 2019 [21]	Responsiveness, empathy	Low empathy, delays	Lower with SERVQUAL gaps	Empathy essential

Predictors and factors associated with patient satisfaction in the emergency department were reported in four studies [13,14,16,19]. Overall doctor satisfaction, perceived waiting time for triage, and meeting patient expectations were significantly associated with patient satisfaction. The perceived quality of healthcare showed a strong correlation with overall doctor satisfaction and fulfillment of patient expectations [13]. One study reported that tangibles, assurance, reliability, responsiveness, and empathy were the main factors influencing patient satisfaction, with tangibles being the most influential and empathy the least influential factor [19]. Another study demonstrated that behavior-related and network-related factors significantly affected patient satisfaction and service perceptions, including physicians’ proximity, ongoing clinical observation, and patient participation in communication networks [14]. In addition, one study reported that shorter emergency department stays and the provision of general, medical, and practical information were associated with increased patient satisfaction, with oral communication and written leaflets being the preferred methods of information delivery [16,17].

Discussion

Recent initiatives in healthcare have increasingly emphasized patient-centered care, individualized services, and active patient involvement in decision-making. Patient satisfaction metrics are widely used to assess the delivery of patient-centered care, and their financial and organizational importance continues to rise. While the relationship between patient satisfaction and clinical outcomes has been examined in various healthcare settings, limited evidence exists regarding the specific determinants of satisfaction among emergency department (ED) patients and how these factors influence their healthcare outcomes [20]. Previous studies have identified timely care, availability of information, staff empathy and attentiveness, and pain management as key elements influencing ED patient satisfaction [21].

Across the included studies, overall patient satisfaction with emergency department services ranged from relatively satisfied to completely satisfied [13,16]. One study reported high satisfaction with medical and general information, while satisfaction with useful information was comparatively lower [16]. These findings highlight variability in patient perceptions of information delivery within the ED setting.

As summarized in Table 2, several positive and negative determinants of patient satisfaction were consistently identified across the included studies. These determinants are discussed in more detail below.

Multiple factors associated with patient satisfaction were consistently reported across the reviewed studies [13,14,16,19]. Overall doctor satisfaction, perceived waiting time for triage, and meeting patient expectations were significantly associated with patient satisfaction [13]. Additional contributing factors included tangibles, assurance, reliability, responsiveness, and empathy, with tangibles showing the strongest association and empathy the weakest [19]. Behavioral and network-related factors were also found to influence patient satisfaction, particularly physicians’ presence, continuous clinical observation, and patient participation in communication networks [14]. Furthermore, shorter ED stays and provision of general, medical, and practical information were associated with increased satisfaction, with oral communication and written leaflets being the preferred information delivery methods [16,17]. Although various information delivery methods have been reported in the literature [22-24], the most favored approaches in the emergency department remain leaflets, videos, and direct communication with healthcare professionals [25,26]. The use of contemporary digital methods such as mobile applications was not widely preferred, with half of the respondents favoring verbal communication [25,26].

The impact of music therapy (MT) on patient satisfaction was also examined. Although no statistically significant differences in satisfaction were reported, patients who received MT experienced notable reductions in stress and pain and expressed willingness to return for further MT sessions [15]. Similar findings have been observed in other hospital settings, where patients exposed to MT demonstrated higher satisfaction scores [27].

Findings from studies conducted in Saudi Arabia revealed moderate levels of patient satisfaction. Satisfaction with doctor and nursing care during the discharge process was reported as 43% and 36%, respectively, in one study [17], and 56% in another [18]. Preferences for waiting time estimates were high, with 70% of participants indicating their importance [17]. Lower satisfaction was reported for translation services, drug information, and pain management. In contrast, higher satisfaction scores were observed for understanding post-discharge symptoms, timely care within 30 minutes, and follow-up inquiries [18]. Longer emergency department stays were associated with lower patient satisfaction, particularly regarding information delivery [28,29]. Waiting time information was consistently identified as the most frequently requested information by patients, and satisfaction was greater when such information was provided [25,26,30,31]. Preferred update intervals ranged between 30 and 41 minutes [25,26].

Limitations

Several limitations should be considered when interpreting the findings of this review. The included studies varied in study design, sample size, and measurement tools, which limits direct comparison between studies. Differences in emergency department environments and healthcare systems also reduce the generalizability of the findings. These limitations underscore the need for more standardized and methodologically robust research to better understand the determinants of patient satisfaction in emergency care settings.

## Conclusions

This systematic review shows that patient satisfaction in emergency departments is influenced by communication quality, the delivery of clear medical and general information, perceived waiting time, and staff responsiveness and empathy. Studies consistently demonstrated that patients who received timely information, practical instructions, and supportive interactions reported higher satisfaction levels. Interventions such as music therapy were also associated with reductions in stress and pain. Although satisfaction levels varied across settings, common areas needing improvement included waiting time management, translation services, and the clarity of medication-related information. Overall, enhancing communication, providing accurate waiting time estimates, and ensuring patient-centered care may significantly improve satisfaction in emergency department settings.
